# A Clinical Prediction Tool for MRI in Emergency Department Patients with Spinal Infection

**DOI:** 10.5811/westjem.2021.5.52007

**Published:** 2021-08-30

**Authors:** Steven R. Shroyer, William T. Davis, Michael D. April, Brit Long, Greg Boys, Sumeru G. Mehta, Sarah F. Mercaldo

**Affiliations:** * Methodist Hospital System, Greater San Antonio Emergency Physicians, San Antonio, Texas; † Methodist Hospital System, Department of Radiology, San Antonio, Texas; ‡ Uniformed Services University of the Health Sciences, Department of Military and Emergency Medicine, Bethesda, Maryland; § 4th Infantry Division, 2nd Brigade Combat Team, Fort Carson, Colorado; ¶ Massachusetts General Hospital, Department of Radiology, Boston, Massachusetts

## Abstract

**Introduction:**

Patients with pyogenic spinal Infection (PSI) are often not diagnosed at their initial presentation, and diagnostic delay is associated with increased morbidity and medical-legal risk. We derived a decision tool to estimate the risk of spinal infection and inform magnetic resonance imaging (MRI) decisions.

**Methods:**

We conducted a two-part prospective observational cohort study that collected variables from spine pain patients over a six-year derivation phase. We fit a multivariable regression model with logistic coefficients rounded to the nearest integer and used them for variable weighting in the final risk score. This score, SIRCH (spine infection risk calculation heuristic), uses four clinical variables to predict PSI. We calculated the statistical performance, MRI utilization, and model fit in the derivation phase. In the second phase we used the same protocol but enrolled only confirmed cases of spinal infection to assess the sensitivity of our prediction tool.

**Results:**

In the derivation phase, we evaluated 134 non-PSI and 40 PSI patients; median age in years was 55.5 (interquartile range [IQR] 38–70 and 51.5 (42–59), respectively. We identified four predictors for our risk score: historical risk factors; fever; progressive neurological deficit; and C-reactive protein (CRP) ≥ 50 milligrams per liter (mg/L). At a threshold SIRCH score of ≥ 3, the predictive model’s sensitivity, specificity, and positive predictive value were, respectively, as follows: 100% (95% confidence interval [CI], 100–100%); 56% (95% CI, 48–64%), and 40% (95% CI, 36–46%). The area under the receiver operator curve was 0.877 (95% CI, 0.829–0.925). The SIRCH score at a threshold of ≥ 3 would prompt significantly fewer MRIs compared to using an elevated CRP (only 99/174 MRIs compared to 144/174 MRIs, P <0.001). In the second phase (49 patient disease-only cohort), the sensitivities of the SIRCH score and CRP use (laboratory standard cut-off 3.5 mg/L) were 92% (95% CI, 84–98%), and 98% (95% CI, 94–100%), respectively.

**Conclusion:**

The SIRCH score provides a sensitive estimate of spinal infection risk and prompts fewer MRIs than elevated CRP (cut-off 3.5 mg/L) or clinician suspicion.

## INTRODUCTION

### Background

Pyogenic spinal infection (PSI), which includes spinal epidural abscess, is an uncommon condition among patients with a common chief complaint of back or neck pain.^1^–^3^ Indeed, back pain is the fifth leading chief complaint among emergency department (ED) patients.^4^ While diagnosing some cases of this infection are simplified by an obvious presentation of back pain and fever, or back pain and intravenous drug use (IVDU), most cases are not easily diagnosed.^1^,^2^,^3^,^5^ The challenge of detecting this uncommon signal from a great deal of background noise can result in diagnostic delay, which can lead to the progression of unrecognized sepsis, permanent neurologic deficit for the patient, and increasing medicolegal risk for the physician.^5^–^10^ Although magnetic resonance imaging (MRI) with gadolinium contrast is 96% sensitive and 93% specific for PSI, it is not an easily administered test. It requires 4–8 hours for test results,^11^ is uncomfortable in some patients, contributes to ED crowding, and is not available at all facilities where back pain is evaluated.^6^,^10^,^12^,^13^

Currently there are no clinical prediction tools to estimate PSI risk,^14^–^17^ no agreement on C-reactive protein (CRP) cut-off levels to indicate imaging,^18^ and no uniform recommendations regarding MRI use.^13^,^19^ Recent publications recommend imaging spine pain patients who have any of the following PSI risk features: historical risk factors; fever; history of fever or progressive neurological deficit,^2^,^6^,^7^,^17^,^20^,^21^ and to consider an alternate diagnosis if none of these are present.^2^,^6^,^20^,^21^

### Goals of this Investigation

We aimed to develop an intuitive risk prediction score using history, physical examination, and CRP measurement that provides a sensitive assessment of the risk of PSI and appropriately recommends MRI.

## METHODS

### Design, Setting, Selection and Population

This was a single-center, observational prospective cohort study conducted in a community ED of 50,000+ adult patients annually in a city of 2.3 million people located in the southwestern United States. Further description of cohort characteristics and methods can be found in earlier publications.^22^,^23^ We enrolled a convenience sample since enrollment required the availability of the primary investigator (PI). We developed a multivariable risk prediction tool in two phases. In the first phase (January 2004–March 2010), we enrolled patients whose emergency physicians suspected they had spinal infection; patients in this phase included both uninfected and PSI patients. From this phase, we selected predictors and derived a risk prediction score. In the second phase (April 2010–August 2018), we followed the same subject identification processes but enrolled only patients with PSI to assess the sensitivity of our prediction tool.

**Population Health Research Capsule**

What do we already know about this issue?

*Pyogenic spinal infection (PSI) is challenging and frequently not diagnosed on the patient’s first visit to a healthcare provider*.

What was the research question?

*Can a sensitive risk prediction tool be derived to identify PSI patients that also avoids overusing MRI resources?*

What was the major finding of the study?

*The novel spine infection risk calculation heuristic score was 100% sensitive and 56% specific for PSI in a derivation cohort and 92% sensitive in a sensitivity assessment cohort*.

How does this improve population health?

*This bedside tool may reduce missed PSI diagnoses, improving morbidity for patients and medical-legal risk for doctors compared to routine clinical evaluation*.

### Eligibility and Data Collection

We considered patients for enrollment if they had back or neck pain (or radicular pain to the limbs or trunk), were ≥ 17 years old, and had no competing diagnoses such as pyelonephritis or pneumonia to explain their pain prior to MRI order. An additional inclusion criterion was that an emergency physician suspected PSI based on the presence of any of the following: historical risk factor^6^; fever (ED temperature ≥ 38°C); recently measured fever before arrival; progressive neurologic deficit (PND), or other factors leading to clinician suspicion such as unusually severe spine pain or bounce-back (return visit following a previous spine-related visit either at our location or another facility). We defined PND as new or worsening weakness, numbness, abnormal reflexes, or urinary incontinence developing within two weeks of the index visit per neurological examination by the PI. We excluded patients who presented less than five days following a spinal surgical procedure;^24^,^25^ if they had a fungal or tuberculous spinal infection; if the diagnosis could not be determined; or if patients without spinal MRI could not be followed in the health record for more than six months after the index visit.

We educated our emergency clinicians on cited PSI risks at the beginning of the study period using illustrative cases. The PI distributed this information by email and at department meetings periodically throughout the study period. Once emergency physicians ordered an MRI or CRP for the purpose of evaluating spinal infection, he or she simultaneously notified the PI. The PI evaluated all patients for enrollment, completed a standardized examination to obtain historical and physical examination findings and available laboratory data, and recorded these on a data collection form. The details of our hospital’s laboratory CRP autoanalyzer and MRI machines are available in prior publications.^22^,^23^ Each subject received usual care, which included counseling discharged patients to return to the ED if they had any symptom progression or development of any new or concerning symptoms. The PI reviewed health records to obtain CRP, imaging interpretations, blood culture results, operative findings, and culture results from surgery and needle aspiration samples.

Our investigation followed the TRIPOD guidelines (transparent reporting of a multivariable prediction model for individual prognosis or diagnosis) for risk prediction model development.^26^ The hospital system’s institutional review board approved the study.

### Outcome Measures

The outcome for our novel risk score SIRCH (spine infection risk calculation heuristic*)* was the presence or absence of PSI, which we defined as the presence of any of the following infections: spinal epidural abscess; vertebral osteomyelitis and/or discitis; paravertebral abscess/infection; paraspinal abscess/infection; or septic facet infection.^3^,^22^ We did not consider isolated psoas muscle infection without another spinal infection to be a PSI. Any of the following confirmed the presence of a PSI: 1) MRI evidence of spinal infection as read by a neuroradiologist; 2) surgical findings of spinal infection on the operative report; or 3) needle aspiration culture results consistent with a spinal infection. The pool of 10 neuroradiologists interpreting images only received patient data to include age, gender, and chief complaint, and we blinded interpreters to the data collected for the study. The MRI imaging was obtained with General Electric Healthcare (Chicago, IL) or Siemens Healthineers (Erlangen, Germany) 1.5 Tesla MRI machines.

Our hospital system used the following MRI protocols: an “MRI with contrast” order included, with slight variation between spinal levels, sagittal and transverse views with T1W, T2W, spin ECHO, T2*GRE and STIR sequences, with additional T1W sagittal and transverse views that included fat suppression following the addition of gadolinium. An MRI order without contrast followed the same protocols except without additional contrast images. Due to the observational nature of our study, not all patients received spinal MRI. Clinical follow-up included a telephone call between two to four weeks after the patient’s index visit, and review of available medical and imaging records for 6–36 months after their index visit to verify that no findings of PSI had developed. We selected this extended follow-up time horizon due to the indolent course of some PSIs. We queried death records at 18 months from index visit on subjects who were lost to follow-up.

### Statistical Analysis

Two investigators double entered all information from the data collection sheets into an Excel database version 14 (Microsoft Corporation, Redmond, WA) and then exported the data into R version 4.0.2 (Foundation for Statistical Computing, Vienna, Austria). We assessed the distributions of infected and uninfected patient characteristics and their differences using the Wilcoxon test for continuous variables and Pearson’s chi-squared test for categorical predictors. We selected candidate predictors and assessed all cases with univariable and multivariable models. We chose a final set of predictors based on those considered to have a biologically plausible association with PSI, while accounting for available degrees of freedom in our model.

We explored several prediction models that included the following: 1) presence of at least one of 10 historical risk factors^6^; 2) fever (defined as ≥ 38°C) on initial ED measurement or reported measurement prior to ED arrival; 3) presence of progressive neurologic deficit; and 4) elevated CRP level. We included CRP in the models as a continuous variable, at varied CRP cut-offs, and used it as a single predictor^23^ (standard laboratory cut-off, 3.5 milligrams per liter [mg/L]). We multiply imputed missing CRP variables using predicted mean matching (1000 imputations), and imputed models were combined using Rubin’s rules.^27^ We report all missing data in Appendix Table 3 and compare complete case, and multiply imputed model performance.

To create a pragmatic model for use in a clinical setting at the patient’s bedside, we then simplified the derived full model by rounding the estimated regression coefficients and assigned these as points to each variable for an easily calculated risk score, understanding there would be a possible trade-off of predictive ability for convenience.^28^ To evaluate each model, we compared the estimated area under the receiver operating characteristic curve (AUROC), calibration intercept, and calibration slope, and we also assessed sensitivity, specificity, accuracy, and positive predictive value (PPV) at the best threshold defined by Youden’s index. We also estimated MRI utilization by calculating the number of MRIs prompted by the SIRCH score. In addition, we evaluated these performance metrics at every possible discrete cut-off of the SIRCH criterion. Finally, we examined our enrollment eligibility’s sensitivity (clinician suspicion) by comparing it to other published PSI screens.^6^,^7^,^9^ We calculated bootstrapped 95% confidence intervals (CI) for each performance metric.

Since existing prevalence data for PSIs in an at-risk population was limited, we based our study size on obtaining at least 10 outcome events for each chosen clinical predictor. A post hoc analysis for sample size, based on an estimated PSI prevalence of 20%, a sensitivity of 95%, and a CI width of 8%, provided an estimated 143 subjects.

## RESULTS

### Baseline characteristics

**Figure 1:**
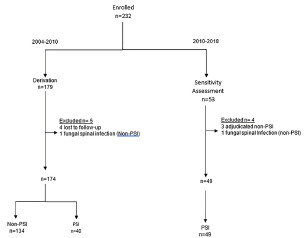
Flow chart of enrolled patients suspected of PSI (derivation) and PSI (sensitivity assessment) PSI, pyogenic spinal infection.

The median age for non-PSI patients was 55.5 (interquartile range [IQR], 38–70), and 30% were male. Of the 89 PSI patients in both phases, the median age was 55 (IQR, 46.7–59.2), 75% were male, 82% had historical risk factors, 37% had a fever or history of measured fever, and 34% had a progressive neurological deficit (Table 1). Of 179 patients enrolled in the derivation phase (Figure 1), we excluded five patients (three lost to follow-up, one fungal infection, and one incomplete follow-up [died without autopsy available]). Thirteen of 134 patients without infection and two of 40 infected patients had no CRP test ordered. Of 134 uninfected patients, 113 (84.3%) had MRI or alternate imaging, and 21 (15.7%) were followed clinically without imaging. Thirty-nine of 40 PSI patients underwent MRI, and confirmation of one infection occurred in the operating room without imaging.

Of 53 patients in the sensitivity assessment cohort (2010–2018), we excluded four (one adjudicated as a superficial post-op infection, one psoas infection without PSI, one retropharyngeal abscess without PSI, and one fungal infection), leaving 49 PSI patients (Figure 1). We imaged 48 patients and confirmed one infection in the operating room without imaging. Six of 49 infected patients had no CRP test ordered. Positive blood culture(s) occurred in 47/82, and a microorganism was isolated in 77/89 infected patients. A total of 189 MRIs and 30 computed tomography images were obtained among the 232 studied subjects.

### Model and Performance

We compared models for statistical performance, discrimination, and calibration and derived the following full model (Table 2):

$$\begin{array}{l} \text{Full PSI Model}=\text{PSI probability}=1/(1+e^{-logit\;function});\text{logit}\\ \text{function}=-5.16+(2.88\times \text{CRP})+(1.6\times \text{RF})+(1.27\times (\text{F or Hx of F}))+(0.84\times \text{PND}). \end{array}$$

We then simplified this model for ease of use at the bedside by rounding regression coefficients to the nearest integer, resulting in the following scoring model to predict PSI probability, called SIRCH (Table 2):

SIRCH score=(3 if CRP≥50 mg/L)+(2 if any RF)+(1 if F or Hx of F)+(1 if PND)

**Figure 2:**
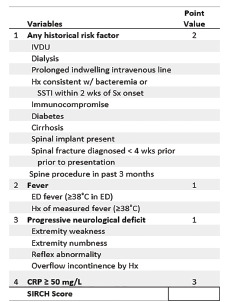
Calculation for spinal infection risk calculation heuristic score. *IVDU*, intravenous drug use; *Hx*, history; *SSTI*, skin and soft tissue infection; *Sx*, symptoms; *wks*, weeks; *ED*, emergency department; CRP, C-reactive protein; mg/L, milligrams per liter.

The SIRCH score (Figure 2) ranged from 0 to 7, and from its ROC we identified a Youden’s cut-off of ≥ 3. We compared the SIRCH score model to three other models (Table 2): full model using CRP continuously; full model with CRP at a cut-off of 3.5 mg/L; and full model with CRP cut-off of 50 mg/L. The SIRCH score had the highest sensitivity and had acceptable MRI utilization, discrimination, and calibration parameters compared to other models (SIRCH score AUROC and calibration plot, Appendix Figure 2 and 3). There was no evidence of a difference in performance metrics of the complete case and multiply imputed models (8.6% missing CRP results). Not shown in the table is the isolated use of the CRP at its standard laboratory cut-off of 3.5 mg/L to decide on imaging. This strategy had a sensitivity of 100% (40/40) and specificity of 22.3% (30/134) and indicated imaging in 144 of the 174 patients, significantly more MRIs compared to 99 (*P* < 0.001) using the SIRCH score.

The SIRCH score predicted PSI at varied criterion cut-offs, as demonstrated in Table 3, (depicted graphically in Appendix Figure 1). In the second phase of our study (2010–2018), the SIRCH score’s sensitivity for PSI declined to 92% (95% CI, 84–98%), while the use of an elevated CRP above the standard cut-off, 3.5 mg/L, was 98% (95% CI, 94, 100%) sensitive.

The median CRP among the 134 uninfected patients was 14 mg/L (IQR, 38–78) — significantly higher than the cut-off for our hospital system’s laboratory standard of 3.5 mg/L. The median CRP for the 40 PSI patients was 120 mg/L (IQR, 69–170) —nearly 50-fold higher. The median CRP for the 49 patients in the second phase was similar to the derivation, 130 mg/L (IQR, 77–182), and consistent with recent studies.^29^,^30^

Of 89 infected patients, 87 had at least one of the following SIRCH criteria: historical risk factor; fever; or progressive neurologic deficit. Although severe pain prompted clinical suspicion of PSI and represented 43% (38/89, Table 1) of PSI patients, other risk features were present in all but two PSI patients. A SIRCH score ≥ 3 identified 85 of 89 (96%) of PSIs overall. In the derivation, the use of historical risk factors as defined by Davis^6^ and CRP above the standard cut-off of 3.5 mg/L had a 90% and 100% sensitivity, respectively. However, SIRCH score specificity (56%) compared favorably to both historical risk factors (37%) and any CRP elevation (22%). The SIRCH score had the best overall combination of high sensitivity (100%), and reasonable utilization, ordering 99 scans to find 40 PSIs (2.48:1).

Characteristics of missed or nearly missed patients with PSIs are shown in Figure 4. The figure indicates that of all 89 infections, only four were missed by SIRCH. Furthermore, a SIRCH score equal to three detected seven infections, but five of these would have been missed if clinicians had used the CRP alone at a cut-off of 50 mg/L to indicate imaging (near-miss). This cautions against an independent use of CRP at this cut-off outside of a multiple variable scoring system. Lastly, the figure indicates “bounce-back” was present in most (10/11) of these patients. And of the 59 bounce-backs, SIRCH would detect all but four of these, implying that 93% (55/59) of these previously missed patients might have had their PSI identified on their prior visit if SIRCH had been available.

**Figure 3:**
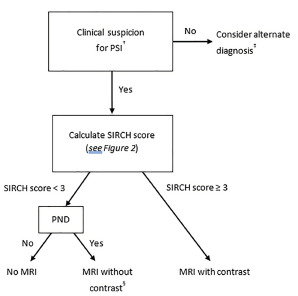
SIRCH algorithm. †Clinical suspicion= enrollment criteria. ‡ Per published recommendations for patients at very low -risk for PSI.^2^,^3^,^19^,^21^ §Patients with a progressive neurologic deficit and a score < 3 require MRI without contrast. To avoid diagnostic delays in high-risk patients who require MRI regardless of CRP result, the authors recommend ordering an MRI with contrast immediately after evaluation and revising to a non-contrast study if SIRCH < 3 with the CRP result. *PND*, progressive neurological deficit; *PSI*, pyogenic spinal infection; *MRI*, magnetic resonance imaging.

Eighty-three percent (25/30) of PSI patients with neurological deficits had no fever to prompt consideration of infection among the 89 spinal infections, highlighting a key circumstance where infection could be overlooked. The algorithm in Figure 3 considers this by using current published recommendations of contrast-enhanced MRI in patients suspected of infection,^3^,^9^,^10^,^12^,^13^,^21^ as indicated by SIRCH score ≥3. For those with a neurological deficit, who are at low risk for infection (SIRCH score of <3), current recommendations indicate that MRI (without contrast) is the appropriate imaging modality.

## DISCUSSION

The imaging prompts, back pain and fever or back pain and IVDU, would have failed to identify a dismal 70% (62/89) of PSIs in our cohort if either prompt were used to decide on MRI. This is in line with the finding by Davis et al that diagnostic failure occurred in 75% (47/63) of PSI patients, and delay in treatment was associated with worse sequelae.^1^ Similar to the Davis study, we found that two-thirds of PSI patients (59/89) in our cohort had a previous medical evaluation for a PSI-related complaint and were not diagnosed with infection (bounce-back). Our study’s derived SIRCH score was sensitive at detecting PSI in our patient population, including the 93% (55/59) of PSI patients not diagnosed on their prior visit, while limiting the number of MRIs compared to CRP use alone.

Our study also supports several other findings from the seminal study by Davis and colleagues.^6^ Both studies are similar in size (89 PSIs vs 86 in Davis), both have a low proportion of infections with fever (19% [17/89] vs 7.3%), and both studies focused on avoidance of MRI in patients at very low risk for infection, which is consistent with current guidelines.^2^,^6^,^20^,^21^,^31^ However, there are four critical differences between the two studies. The study by Davis et al had a high prevalence of IVDU compared to the current study (60% vs 4.5%); Secondly, the Davis screen, using risk factors^6^ only, was 82% (72/89) sensitive for PSI, compared to a SIRCH sensitivity of 96% (85/89). Third, the SIRCH algorithm considers progressive neurologic deficit a risk factor to be used in screening for PSI and recommends a contrast MRI for patients with a SIRCH score of ≥ 3, whereas the Davis protocol considers a CRP unnecessary in the case of neurologic deficit. However, adding contrast to the MRI in this instance avoids the following pitfall: Most patients presenting with a PSI in our study did not have a fever, and likewise, 83% (25/30) who had a neurological deficit did not have a fever either. Clinicians not actively looking for PSI may not suspect infection in this group and imaging an infected patient without contrast may lead to a missed PSI or an equivocal reading. This circumstance may prompt a neuroradiologist to recommend repeating the MRI but with the addition of contrast, which adds another 4–8 hours^11^ to the ED evaluation and the patient’s time in the ED. The fourth and final difference between the two studies is that Davis recommends using a CRP level after screening as the primary arbiter in PSI prediction, whereas our study derived a CRP cut-off and used the CRP as one of four elements in a scoring model to predict PSI.

Authors have recommended various methods to improve clinical recognition of PSI, including the use of red flags.^7^,^20^,^21^,^32^ However, the red flags as defined by Bhise^13^ lacked adequate sensitivity (69%) for clinical use in our patient population. Inconsistencies in published guideline recommendations and imprecise risk factor definitions^14^–^16^,^19^ may be responsible for incomplete adoption of any single recommendation for imaging decisions. The resulting indifference to their use may play a role in the high diagnostic failure rate cited by Bhise.^7^

**Figure 4:**
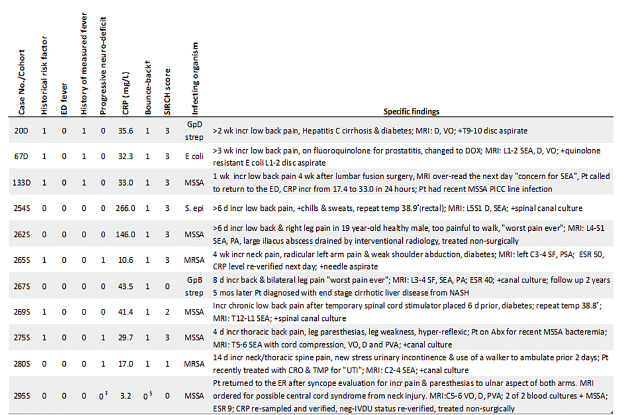
Patient characteristics in all SIRCH score misses or near-misses. 1= present, 0= absent. †Bounce-back= a prior ED/clinic visit related to current visit; ‡MRI ordered based on suspicion of central cord syndrome but adjudicated not a progressive neurological deficit. §Not considered a bounce-back since patient’s first visit unrelated to PSI; *PSI*, pyogenic spinal infection; *SEA*, spinal epidural abscess; *VO*, vertebral osteomyelistis; *D*, discitis, *PVA*, paravertebral abscess; *PSA*, paraspinous abscess; *SF*, septic facet; *PA*, psoas abscess; *Abx*, antibiotics; *CRO*, ceftriaxone; *DOX*, doxycycline; *TMP*, trimethoprim-sulfamethoxazole; *SSTI*, skin and soft tissue infection; *MSSA*, methicillin sensitive *Staphylococcus aureus*; *MRSA*, methicillin resistant *Staphylococcus aureus*; *S epi, Staphylococcus epidermidis*; *d*, day; *wk*, week; *incr*, increasing; *ESR*, erythrocyte sedimentation rate; *NASH*, non alcoholic steatohepatitis; *Pt*, patient.

Clinician specificity for PSI is also poor, with studies finding between 15–30 MRIs are ordered to find one infection.^13^,^19^ The use of MRI is an important factor since its lengthy turnaround time of 4–8 hours^11^ has been cited as “contributing to ED overcrowding.”^13^ Of the 134 uninfected patients in our derivation cohort, the SIRCH score would reduce the number of unnecessary imaging by 75 compared to clinician suspicion, while the Davis risk factors and any CRP elevation would reduce it by 50 and 30, respectively. And although CRP was 100% (40/40) sensitive for the infection, its specificity was considered unacceptable for clinical use (144 scans to find 40 infections), and given the ubiquity of back pain, CRP testing in unselected patients would likely result in increased MRI overuse. Various CRP cut-offs have been recommended in the literature. We selected a cut-off unique to our at-risk spine pain cohort to maximize its accuracy for this population, and clinicians using this cut-off should be aware of instances in which the CRP may be lower than our cut-off in PSI patients, especially those with cirrhotic liver disease or concurrent antibiotic use (5 of the 11 misses or near-misses in Figure 4).^23^–,^25^ In this study, the presence of other risk variables heightened suspicion of infection, which maintained our high sensitivity for these cases.

Our study shows SIRCH is sensitive for the clinical detection of PSI and would limit the number of scans compared to using CRP after screening for PSI. However, it can be noted that reducing the number of MRIs in our cohort by 75 over our long study period may not have had a large impact on ED crowding. Nonetheless, the impact is likely to be magnified with any attempt to improve the sensitivity for this uncommon and challenging diagnosis without a method in place to limit false positives, leading to more overuse of MRI resources, not less.

## LIMITATIONS

This study’s single-center design may restrict the generalizability of our findings. Our sample was not consecutive and only included patients when spinal infection was clinically suspected. Our convenience sample’s high PSI prevalence may subject our study to spectrum bias, which could result in overestimating the SIRCH score’s accuracy. Additionally, our enriched sample could overestimate the SIRCH score’s MRI utilization benefits (fewer false positives) compared to lower prevalence populations. The low prevalence of IVDU in our sample may restrict generalizability to settings with more PSI secondary to drug injection.

Although blinding clinicians to the CRP results could have reduced potential work-up bias, this was inconsistent with the observational nature of our study. However, we believe the risk of this bias was minimal based on the following: there is no widely accepted cut-off recommendation for CRP use in predicting PSIs; no diagnostic accuracy study validating its value in PSI;^18^ and the test is widely known to have poor specificity. This knowledge may have led to fewer CRP test orders in PSI patients as the study progressed (CRP not ordered in two in the derivation and six in the second phase). Despite this, there is potential for this bias to overstate the accuracy of our prediction score.

Not all patients were evaluated using a single reference standard (MRI); however, two investigators reviewed all radiology reports and images and confirmed equivocal MRI reads with culture and operating reports. We defined PSI precisely using the most contemporary nomenclature,^3^,^34^ and the 21 uninfected cases that had no MRI were followed clinically for a prolonged duration to verify no occurrence of infection. We contend that this protocol provided a robust reference standard. Despite telephone follow-up, extended health record follow-up, and death records search, the potential for improper classification of missed infections exists. The study’s 14-year duration may have subjected it to temporal bias due to increased MRI availability or improved clinician confidence in selecting and diagnosing spinal infection over this long period. Over this time, clinicians may have depended less on well-known high-risk features of PSI and more on acquired expertise, leading to the identification of more PSI patients in the second half of the study who had no fever, no historical risk factors, and who had more missing CRP orders.

A single, experienced emergency physician collected the study data, so this prevents measurement of interobserver variability. We mitigated this by using the most objective variables available and those with previously published measurements of interobserver variability.^35^ A small number of enrolled patients were later found to have posterior lower lobe pneumonia or pyelonephritis as the cause of their back pain. Had these conditions been recognized prior to spinal MRI order, the study would have resulted in greater CRP and SIRCH score specificities. Finally, our study’s small size required us to combine several variables into composite variables, possibly concealing the strength of crucial individual risk factors.

## CONCLUSION

In 2020 Galliker et al wrote, “To date, there has been no risk prediction tool to assist [emergency] physicians in assessing patients with low back pain.”^14^ The SIRCH score was 100% sensitive for pyogenic spinal infection and prompted fewer MRIs than clinician suspicion or CRP use in our derivation cohort but was less sensitive in the second phase (92%) compared to CRP (98%). This bedside scoring system, using clinical findings and CRP to inform spinal MRI decisions, requires external validation in other ED settings prior to clinical use.

## Supplementary Information



## Figures and Tables

**Table 1:** Patient characteristics in 223 patients suspected of pyogenic spinal infection.

	Derivation					Sensitivity assessment	
		
Potential predictor variables	No infection N=134	%	PSI N=40	%	P- value	PSI N=49	%
Mean age, (IQR); years	55.5	(38–70)	51.5	(42–59)	0.577	57	(51–64)
Gender, male	40	30%	30	75%	<0.001	32	65%
Historical risk factors	84	63%	36	90%	0.001	37	76%
IVDU	0	0%	3	7.5%	0.001	7	14%
Dialysis	4	3.0%	3	7.5%	0.202	2	4.0%
Prolonged indwelling IV (PICC, temporary dialysis catheter etc.)	0	0%	4	10%	<0.001	7	14%
Hx consistent w/bacteremia or SSTI within 2 wks of Sx onset	3	2.4%	15	38%	<0.001	13	27%
Immunocompromise	4	3%	2	4.1%	0.54	2	4.0%
Diabetes	39	29%	17	43%	0.112	19	39%
Cirrhosis	0	0%	3	7.5%	0.001	4	8.2%
Spinal implant present (spinal pump, cord simulator, etc.)	7	5.2%	0	0%	0.14	2	4.1%
Spinal fracture recently diagnosed (< 4 wks prior to presentation)	0		0			0	
Spine procedure in past 3 months	45	34%	14	35%	0.868	15	31%
Fever in ED or Hx or measured fever	30	22%	23	58%	<0.001	10	20%
ED fever (≥38°C in ED)†	18	13%	12	30%	0.017	5	10%
Hx of measured fever (≥38°C) and no ED fever	12	9%	11	28%	0.002	5	10%
Any new (spine-related) neurological deficit	28	21%	15	38%	0.033	15	31%
New extremity weakness	21	16%	9	21%	0.316	8	16%
Overflow incontinence by Hx	8	6.0%	8	20%	0.007	7	14%
Extremity numbness	14	10%	6	15%	0.430	4	8.2%
Reflex abnormality	5	3.7%	5	13%	0.037	4	8.2%
Bounce-back within 2 wks	NA	NA	25	63%		34	69%
Temperature, median, (IQR); °C	36.8	(36.3–7.2)	37.3	(36.7–38.2)	0.01	36.8	(36.6–37.4)
Mean arterial pressure, (IQR); mm Hg	98.3	(88.2–109)	96.0	(81.3–107)	0.161	97	(86.3–106)
HR, median, (IQR); beats/minute	86	(74–103)	94	(80–107)	0.121	94	(84–103)
WBC, median, (IQR); cells/μL	8.8	(7.2–11.5)	11.1	(9.1–13.2)	0.001	12.1	(8.9–15)
CRP, median, (IQR); mg/L	14.0	(3.8–78)	120	(69–170)	<0.001	130	(76.6–182)
Spine pain character							
Worst pain ever	15	11%	9	23%	0.070	17	35%
Intermittent radicular	23	17%	2	5.0%	0.008	12	24%
Constant severe radicular	30	22%	7	18%	0.561	19	39%
Intermittent or constant radicular	51	38%	9	23%	0.070	27	56%
Unable to sit up independently due to pain	30	22%	15	38%	0.044	23	47%
Unable to ambulate due to pain	31	23%	16	40%	0.036	6	12%

† ED fever = first temperature obtained in the ED ≥ 38° C (100.4° F).

*PSI*, pyogenic spinal infection; *ED*, emergency department; *IVDU*, intravenous drug use; *PICC*, peripherally inserted central line; *SSTI*, skin and soft tissue infection; *NA*, not available; *wks*, weeks; *Sx*, symptoms; *Hx*, history; *HR*, heart rate; *IQR*, interquartile range; *mm Hg*, millimeters mercury; *μL*, microliters; *mg*, milligrams.

**Table 2:** Multivariable full prediction models and SIRCH score.

Model variables	Model, continous CRP	Full model, CRP ≥ 3.5	Full PSI model, CRP ≥ 50	SIRCH, CRP ≥ 50
Intercept	−4.32 (−5.81, −2.84)	−8.23 (−55.19, 38.72)	−5.16 (−6.92, −3.40)	
CRP	0.01 (0.01, 0.02)	4.72 (−42.25, 51.69)	2.88 (1.62, 4.15)	3
Any risk factor	1.78 (0.49, 3.06)	1.64 (0.48, 2.80)	1.60 (0.31, 2.89)	2
Fever	1.20 (0.28, 2.11)	1.68 (0.85, 2.51)	1.27 (0.33, 2.20)	1
Any neuro-deficit	0.80 (−0.18, 1.79)	1.22 (0.34, 2.11)	0.84 (−0.17, 1.85)	1
Performance				
AUC	0.867 (0.813, 0.922)	0.778 (0.704, 0.852)	0.886 (0.839, 0.934)	0.877 (0.829, 0.925)
Cal int	0.034 (−0.489, 0.587)	0.008 (−0.526, 0.554)	0.039 (−0.440, 0.533)	−5.229 (−7.136, −3.769)
Cal slope	1.032 (0.705, 1.418)	1.002 (0.628, 1.434)	1.027 (0.719, 1.411)	0.938 (0.652, 1.295)
Threshold	−1.214 (−2.066, −0.727)	−0.670 (−1.851, −0.418)	−1.222 (−2.507, −0.794)	3.000 (3.000, 3.000)
Sensitivity	0.850 (0.725, 1.000)	0.725 (0.525, 0.900)	0.950 (0.850, 1.000)	1.000 (1.000, 1.000)
Specificity	0.813 (0.552, 0.918)	0.731 (0.597, 0.866)	0.754 (0.597, 0.851)	0.560 (0.478, 0.642)
Accuracy	0.816 (0.655, 0.891)	0.736 (0.632, 0.810)	0.793 (0.690, 0.862)	0.661 (0.598, 0.724)
PPV	0.569 (0.400, 0.732)	0.450 (0.348, 0.583)	0.529 (0.426, 0.648)	0.404 (0.364, 0.455)
MRIs indicated†	61/174	66/174	70/174	99/174
Sensitivity assessment ‡	0.610 (0.470, 0.760)	0.310 (0.180, 0.450)	0.710 (0.590, 0.840)	0.920 (0.840, 0.980)

† MRIs indicated= Number of patients identified as positive by the model recommending spinal MRI to evaluate for PSI.

‡ Sensitivity assessment= second phase, infection only cohort, 2010–2018.

*SIRCH*, spine infection risk calculation heuristic; *PSI*, pyogenic spinal infection; *CRP*, C-reactive protein; *AUC*, area under the curve; *PPV*, positive predictive value; *MRI*, magnetic resonance imaging.

**Table 3:** Probability of pyogenic spinal infection and number of magnetic resonance images indicated from SIRCH* score cut-off criterion.

Performance	0	1	2	3	4	5	6	7
Sensitivity	1.00 (1.00, 1.00)	1.00 (1.00, 1.00)	1.00 (1.00, 1.00)	1.00 (1.00, 1.00)	0.93 (0.83, 1.00)	0.88 (0.77, 0.98)	0.60 (0.45, 0.75)	0.13 (0.03, 0.23)
Specificity	0.00 (0.00, 0.00)	0.23 (0.15, 0.29)	0.31 (0.23, 0.38)	0.56 (0.48, 0.64)	0.73 (0.66, 0.81)	0.77 (0.69, 0.84)	0.87 (0.81, 0.93)	0.99 (0.96, 1.00)
Accuracy	0.23 (0.23, 0.23)	0.40 (0.35, 0.45)	0.47 (0.41, 0.52)	0.66 (0.60, 0.72)	0.78 (0.71, 0.83)	0.79 (0.74, 0.85)	0.81 (0.75, 0.86)	0.79 (0.76, 0.82)
PPV	0.23 (0.23, 0.23)	0.28 (0.26, 0.30)	0.30 (0.28, 0.33)	0.40 (0.36, 0.46)	0.51 (0.44, 0.59)	0.53 (0.46, 0.62)	0.59 (0.47, 0.71)	0.73 (0.33, 1.00)
MRIs indicated	174/174	145/174	133/174	99/174	73/174	66/174	41/174	7/174
Sensitivity assessment†	1.00 (1.00, 1.00)	0.960 (0.90, 1.00)	0.94 (0.86, 1.00)	0.92 (0.84, 0.98)	0.84 (0.73, 0.94)	0.710 (0.59, 0.84)	0.270 (0.14, 0.39)	0.02 (0.00, 0.06)

† Sensitivity assessment = 2nd phase cohort 2010–2018, infection only; SIRCH score uses all possible threshold cutpoints (0–7), multiply imputed models.

* *SIRCH*, spinal infection risk calculation heuristic.
