# Supplementary material for: A Clinical Prediction Tool for MRI in Emergency Department Patients with Spinal Infection
Source: West J Emerg Med. 2021 Aug 30;22(5):1156–66. doi: 10.5811/westjem.2021.5.52007 (PMC8463051; doi:10.5811/westjem.2021.5.52007)
Supplement: Supplementary file 1 [file wjem-22-1156-s001.docx]

**Appendix**

Appendix, Variable definitions…………………………………………………………………………………………..Page 1, 2

Appendix, Table Missing Data Analysis……………………………………………………………………………. Page 3

Appendix, Figure 1. Probability of PSI at varied SIRCH score criterion cut-offs…………………..Page 4

Appendix, Figure 2. Calibration Plot…………………………………………………………………………………..Page 5

Appendix, Figure 3. Area under the receiver operator curve (AUROC) ……………………………..Page 6

**Appendix 1.** Definitions: Historical risk factor variables were defined according to commonly accepted emergency medicine definitions and chosen based on the following plausibilities: 1. A variable’s association with bloodstream infection, resulting in bacterial spinal seeding; 2. A variable’s increased susceptibility to being seeded by a bloodstream infection (e.g., spinal implants due to biofilm, spinal fracture hematoma, or recent spinal surgical procedures); or 3. A variable’s potential for local infection to cause a spinal neurologic deficit.

1. Skin and soft tissue infections (SSTI) or bacteremic condition: Skin abscesses/cellulitis or any bacterial infection that may cause bacteremia (e.g., MRSA abscess, peritonsillar abscess, perirectal abscess, pneumonia, osteomyelitis, pyelonephritis, endocarditis, etc.) present within two weeks of onset of spine pain.
2. Intravenous drug use (IVDU): Patient report of prior history of intravenous drug use.
3. Dialysis: Use of (hemodialysis for renal failure) by patient report or medical record review.
4. Chronic indwelling intravenous catheter: Intravenous lines such as a mediport, PICC line, or central venous line, in place longer than 7 days.
5. Immunocompromise: Patient who is undergoing treatment with cytotoxic chemotherapy, immunomodulators for organ transplantation, who has had a splenectomy, or who has a hematologic malignancy (e.g., multiple myeloma and chronic lymphocytic leukemia) may have a susceptibility to bacteremia. Patients treated with TNF α blockers such as adalimumab, etanercept, infliximab, etc., were considered potentially immunocompromised.
6. Diabetes: Patients reporting a history of diabetes or who are taking diabetic medication.
7. Cirrhotic liver disease: Patients who report a diagnosis of cirrhotic liver disease (e.g., hepatitis C, alcoholic, NASH, etc.).
8. An intraspinal device: Spinal device or hardware that has been in place for more than three months, such as a spinal cord stimulator, morphine or baclofen pump, lumboperitoneal shunt, etc.
9. Vertebral fracture: Recently diagnosed vertebral fracture(s) prior to PSI symptoms.
10. Recent spine procedure: Any surgical spine procedure within 3 months of presentation. Examples include minimally invasive tubular retractor surgery, discectomy, myelography, discogram, vertebral body fusion, lumbar puncture, kyphoplasty, vertebroplasty, epidural steroid injection, obstetric epidural anesthetic procedure, spinal cord stimulator placement etc. Patients presenting to the ED less than 5 postoperative days are excluded since the CRP may be elevated without infection.^23,24,33^
11. Progressive neurological deficit (spinal cord or nerve injury): Any of the following findings that have developed in the two weeks preceding the patient's presentation: radicular pain, paresthesias, or positional paresthesias that are in a dermatomal distribution, bilateral radicular paresthesias or pain, extremity weakness, overflow incontinence or urinary retention by history or by post-void residual measurement ≥ 500 mLs, abnormal reflexes including extremity hyperreflexia or areflexia, abnormal Babinski's or Hoffmann's reflexes, + Spurling's test or straight leg raising test for radicular pain, + cross straight leg raising test, + Lasegue's sign. A neurological syndrome such as central cord syndrome and cauda equina syndrome are considered to be neurological deficits.
12. Bounce-back: A patient who returns to the ED for back/neck pain or a PSI related symptom who was not diagnosed on the prior visit with PSI. PSI related symptoms may include a new neurologic symptom such as incontinence, radicular pain or paresthesias, new falls for suspected leg weakness, the progression of pain intensity, or the progression of radicular pain from unilateral to bilateral. For patients presenting to the ED ≥ 5 days after a spinal procedure, the surgical encounter does not count as a prior visit.
13. Red Flags: Five of nine red flags listed by Bhise et al. ^7^ and Singleton et al. ^19^ are associated with spinal infection and include the following: 1. unexplained fever, 2. focal neurologic deficit with progressive or disabling symptoms, 3. active infection, 4. immune suppression, 5. intravenous drug use (The four red flags unrelated to spinal infections include the following: use of steroids, weight loss, back pain longer than six weeks, and history of cancer).

**Appendix Figure 1. Probability of PSI at varied SIRCH score criterion cut-offs**


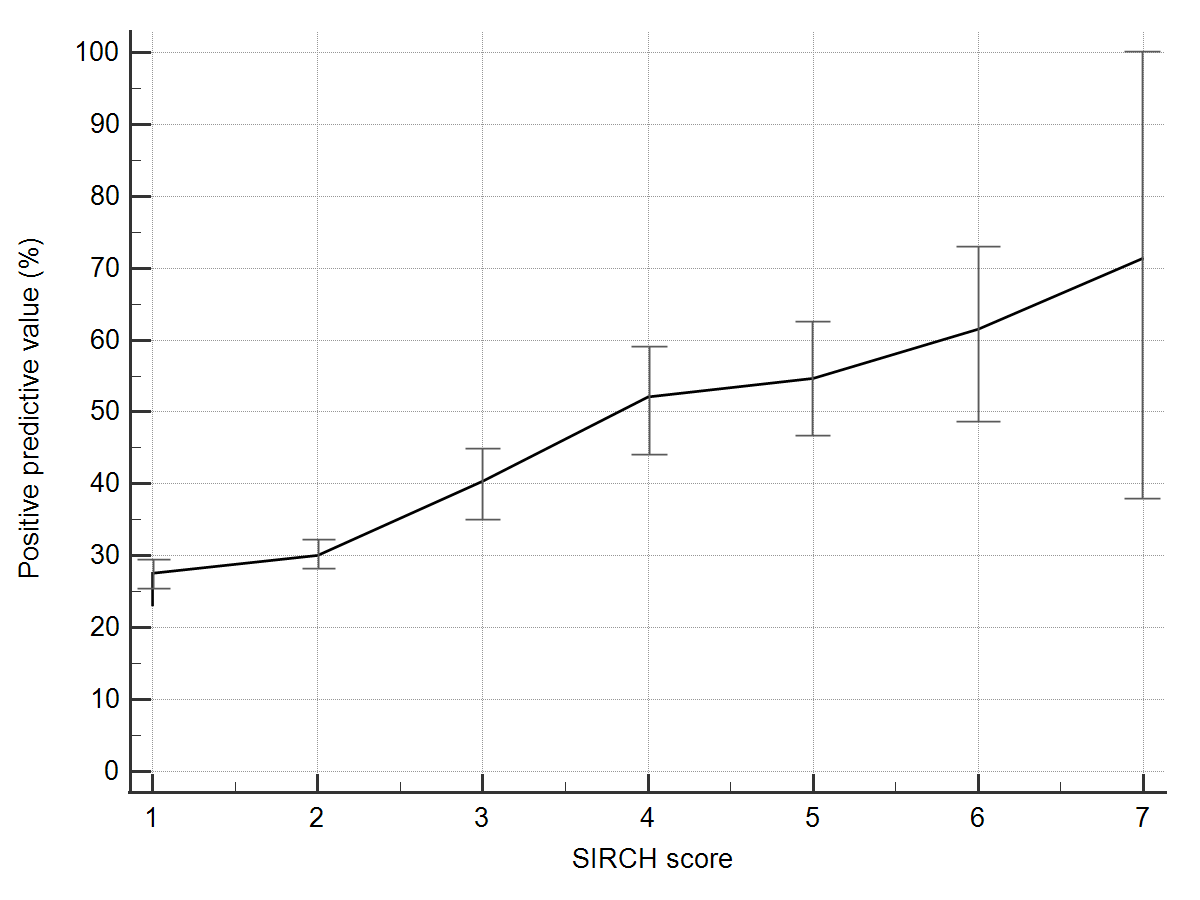


**Appendix Figure 2. Calibration Plot**

Predicted Probability

0.0

0.2

0.4

0.6

0.8

1.0

0.0

0.2

0.4

0.6

0.8

1.0

Actual Probability

Ideal

Logistic calibration

Nonparametric

Dxy

C (ROC)

R2

D

U

Q

Brier

Intercept

Slope

Emax

E90

Eavg

S:z

S:p

0.773

0.886

0.496

0.390

−0.011

0.402

0.115

0.039

1.027

0.095

0.067

0.029

0.326

0.745

**Appendix Figure 3. Area under the ROC**

### Comparing Risk Models to Predict PSI

Sensitivity

0.0

0.2

0.4

0.6

0.8

1.0

0.0

0.2

0.4

0.6

0.8

1.0

AUC (95 CI)

CRP Only

Model w/ CRP>=50

SIRCH w/ CRP>=50

0.85 (0.80,0.91)

0.89 (0.84,0.93)

0.88 (0.83,0.93)

False positive rate
